# Discovery, characterization and mechanism of a *Microbacterium* esterase for key *d*-biotin chiral intermediate synthesis

**DOI:** 10.1186/s40643-024-00776-2

**Published:** 2024-06-16

**Authors:** Xinjia Li, Haoran Yu, Shengli Liu, Baodi Ma, Xiaomei Wu, Xuesong Zheng, Yi Xu

**Affiliations:** 1 School of Chemical and Environmental Engineering, Shanghai Institute of Technology, 100 Haiquan Road, Shanghai, 201418 China; 2 Xianghu Laboratory, Hangzhou, 311231 China; 3 Institute of Bioengineering, College of Chemical and Biological Engineering, Zhejiang University, Hangzhou, Zhejiang 310027 China; 4 Shandong Lonct Enzymes Co., Ltd, Linyi, 276400 China; 5 School of Perfume and Aroma Technology, Shanghai Institute of Technology, 100 Haiquan Road, Shanghai, 201418 China

**Keywords:** Genome mining, Esterase, Stereoselective hydrolysis, *d-*Biotin

## Abstract

Esterases are crucial biocatalysts in chiral compound synthesis. Herein, a novel esterase EstSIT01 belonging to family V was identified from *Microbacterium chocolatum* SIT101 through genome mining and phylogenetic analysis. EstSIT01 demonstrated remarkable efficiency in asymmetrically hydrolyzing meso-dimethyl ester [Dimethyl cis-1,3-Dibenzyl-2-imidazolidine-4,5-dicarboxyate], producing over 99% yield and 99% enantiomeric excess (*e.e.*) for (4*S*, 5*R*)-monomethyl ester, a crucial chiral intermediate during the synthesis of *d*-biotin. Notably, the recombinant *E. coli* expressing EstSIT01 exhibited over 40-fold higher activity than that of the wild strain. EstSIT01 displays a preference for short-chain *p*-NP esters. The optimal temperature and pH were 45 °C and 10.0, with *K*_m_ and *k*_cat_ values of 0.147 mmol/L and 5.808 s^− 1^, respectively. Molecular docking and MD simulations suggest that the high stereoselectivity for meso-diester may attribute to the narrow entrance tunnel and unique binding pocket structure. Collectively, EstSIT01 holds great potential for preparing chiral carboxylic acids and esters.

**Supplementary Information:**

The online version contains supplementary material available at 10.1186/s40643-024-00776-2.

## Introduction

Recently, esterases have been widely studied as important biocatalysts for enantioselective synthesis of optically pure chiral carboxylic acids and derivatives. Their exceptional stability under harsh conditions and excellent enantioselectivity make them highly valuable in a wide range of applications, particularly in the food, pharmaceutical, and environmental bioremediation industries (Bhatt et al. 2020, 2021; Bornscheuer 2002; Cavalcante et al. 2021; Dou et al. 2022; Le et al. 2022; Nguyen et al. 2011; Oliveira et al. 2019; Romano et al. 2015; Schreck and Grunden 2014). Esterase (EC 3.1.1.1) and lipase (EC 3.1.1.3) are commonly referred to as lipolytic enzymes. Generally, lipases are capable of hydrolyzing water-insoluble and long-chain esters, while esterases have primarily been considered as enzymes that act on short-chain esters. Unlike many enzymes, esterase (EC 3.1.1.1) could hydrolyze or form ester bonds, without requiring cofactors or metal ions. Most esterase possess the characteristic α/β hydrolase fold structure, where strands in a central β-sheet are connected by α-helixes. Additionally, a hydrophobic pocket surrounds the active catalytic triad composed of Ser-His-Asp/Glu (Arpigny et al. 1999; Gao et al. 2019; Tahir et al. 2020). Due to the difficulty and cost involved in obtaining esterases from animals and plants, microbial sources have become the predominant choice for industrial applications. Microorganisms such as *Candida*, *Pseudomonas*, *Actinobacteria* and *Aspergillus niger* have been extensively explored as potential sources of esterases (Cieśliński et al. 2007; Jeon et al. 2009; Ma et al. 2013; Papanikolaou et al. 2023; Staudt et al. 2022; Wei et al. 2013; Zhang et al. 2014).

*d-*Biotin plays a vital role in maintaining the balance of human metabolism, including carbohydrate metabolism, amino acid metabolism and lipid metabolism. It finds extensive applications in various fields such as medicine, functional food and animal husbandry, leading to a significant global market demand. Traditionally, *d-*Biotin has been produced through chemical synthesis in the industry (Chen et al. 2005). However, these methods have several environmental drawbacks, making enzymatic synthesis a more environmentally friendly alternative. Currently, the enzymatic asymmetric synthesis of optically pure (4*S*,5*R*)-monoester [(4*S*,5*R*)-2-imidazolone-4,5-dicarboxylic acid mono-ester] holds a pivotal role in the production of *d*-biotin, and this approach, along with microbial synthetic biology techniques, has emerged as a noteworthy and environmentally friendly method for biotin synthesis (Chen et al. 2005, 2011; Lazar et al. 2017; Noguchi et al. 2013; Xiao et al. 2020). However, the previously reported enzymes were either expensive porcine liver esterases or low-activity enzymes derived from natural sources, which limited the application of enzymatic methods in the synthesis of (4*S*,5*R*)-monoester.

Shotgun approaches are commonly employed for the discovery of novel enzymes (Li et al. 2008; Zhang et al. 2009). The experimental workflow typically involves the extraction and fragmentation of genomic or metagenomic DNA, followed by the generation of fragments of desired length through electrophoresis. These fragments are then ligated with vectors and transformed into *E. coli*, and the resulting colonies are screened using specific substrates (Dou et al. 2020; Lee et al. 2021). However, if a novel enzyme with specific activity is localized to a particular microorganism, the experimental efforts involved in shotgun approaches can be immense. Benefiting from the development of high-throughput sequencing, genome mining has emerged as a widely adopted strategy for the discovery of new enzymes. Additionally, bioinformatics and other rational methods can be employed to identify novel functional enzymes. For example, Meinert et al. utilized SSFE (Sequence-Structure-Function-Evolution) strategy to discover novel chalcone isomerases for enzymatic synthesis of (*S*)-flavanones (Meinert et al. 2021). Chen et al. employed big data mining, rational modification, and ancestral sequence reconstruction to obtain multiple xylose isomerases for biorefinery applications (Chen et al. 2023). Liu et al. employed genome mining to identify a Baeyer-Villiger monooxygenase from *Cupriavidus basilensis* for the asymmetric synthesis of (*R*)-lansoprazole and other pharmaco-sulfoxides (Liu et al. 2021). These methods, coupled with genome mining, can help narrow down the candidates when searching for target enzymes with specific functions (Ilmén et al. 2015; Zhu et al. 2015).

**Scheme 1:**
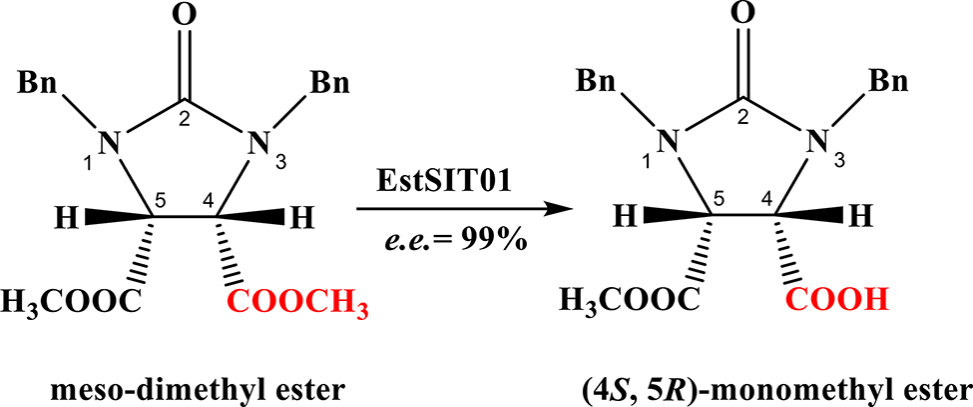
The stereoselective hydrolysis of *meso*-dimethyl ester by EstSIT01

In our previously published paper, we reported on the selective immobilization of crude recombinant esterase from *Microbacterium chocolatum* SIT101 using two types of nano-materials. Both immobilized enzymes demonstrated higher stability compared to their free counterparts and could be reused for more than 10 cycles without significant loss of enzyme activity in the synthesis of *d*-biotin chiral intermediates, highlighting the potential applications of these immobilized enzymes (He et al. 2021; Wei et al. 2022). However, the discovery of the gene encoding the *Microbacterium* esterase, the enzymatic characterization of the purified esterase, and the mechanistic analysis of its catalytic asymmetric synthesis of chiral biotin intermediates have not been reported. In this paper, we mainly describe how we identified the esterase (EstSIT01, Scheme 1) from the wild *Microbacterium chocolatum* SIT101 through genome mining and phylogenetic analysis. We also investigated the enzymatic properties of the purified recombinant EstSIT01, including temperature, pH, substrate specificity and kinetics. Additionally, we employed molecular dynamics simulations to gain a better understanding of the mechanism of EstSIT01 for stereoselective hydrolysis of the substrate (meso-dimethyl ester). The findings of this study will further elucidate the enzymatic properties and catalytic reaction mechanism of *Microbacterium* esterase, providing experimental and theoretical references for further molecular modifications of this enzyme.

## Materials and methods

### Microorganisms, plasmids and chemicals

*Microbacterium chocolatum* SIT101 (CGMCC 4436, Beijing, China) was preserved in our laboratory. *E. coli* BL21 (DE3) and pET-21a (+) (Sangon Co., Ltd, Shanghai, China) were used as the host and vector, respectively. Restriction enzymes were purchased from Takara Biomedical Technology Co., Ltd (Beijing, China). Ni-NTA column was from Changzhou Smart-Lifesciences Biotechnology Co., Ltd. (Changzhou, China). Oligonucleotide primers and electrophoresis reagents were obtained from Generay Biotechnology Co., Ltd (Shanghai, China). Meso-diester and (4*S*, 5*R*)-monoester were provided by Zhejiang Shengda Bio-pharm Co., Ltd. All other chemicals used in the assay were purchased from general commercial suppliers and used without additional purification.

### High-throughput sequencing, de Novo assembly and genome mining

The complete genome of *Microbacterium chocolatum* SIT101 was sequenced using Illumina HiSeq 4000 and PacBio RS II sequencing platforms by BGI (Shenzhen, China). Genome mining was performed to identify potential enzymes based on the complete genome information of SIT101. Sequence alignment and phylogenetic tree analysis were both conducted using ClustalX2.

### Cloning, overexpression and purification

The genomic DNA extracted from *Microbacterium chocolatum* SIT101 was used as a template for PCR amplification of the predicted enzyme genes. The primers (Table S1), with the underlined sequences representing the restriction enzyme cutting sites, were used for PCR. SignalP 4.1 was utilized for the prediction of the signal peptide sequence (Petersen et al. 2011). The PCR fragments were then ligated into the pET21a vector, which had been previously digested with the same restriction enzymes. The resulting recombinant plasmid, pET21a-EstSIT01, containing a 6×His tag at the carboxyl terminal, was transformed into *E. coli* BL21(DE3) cells.

The transformed *E. coli* BL21(DE3) cells carrying the recombinant plasmid were cultured in LB medium supplemented with 100 µg/mL ampicillin at 200 rpm and 37 °C. Once the OD_600_ reached 0.6 ~ 0.8, the growth temperature was reduced to 20 °C, and IPTG (isopropyl-β-D-thiogalactopyranoside) was added to a final concentration of 0.2 mmol/L to induce protein overexpression. After 16 h of induction, the cells were harvested by centrifugation at 7000 rpm for 15 min. The sediment was then resuspended in lysis buffer (50 mmol/L NaH_2_PO_4_, 300 mmol/L NaCl, 10 mmol/L imidazole, pH 8.0) and subjected to ultrasonication on ice. The cell lysate was centrifuged at 10,000 rpm for 30 min at 4 °C, and the resulting supernatant was loaded onto a Ni-NTA Beads 6FF column (5 mL) pre-equilibrated with lysis buffer. The enzyme was eluted using the 250 mmol/L imidazole buffer. The purity of the collected fractions was assessed by SDS-PAGE, and the fractions containing the pure protein were concentrated using a 10 kDa cut-off membrane (Millipore, Billerica, USA) through ultrafiltration. This step helped remove impurities such as salts, small molecule compounds, polypeptides, and macromolecules below 10 kDa. Solution replacement with PBS (0.2 mol/L, pH 8.0) was performed by low-speed centrifugation to replace the high concentration of imidazole solution. The protein concentration was quantified utilizing the Bradford method, and subsequently adjusted to a concentration of 1 mg/mL through dilution.

### Determination of enzyme properties of EstSIT01

Enzymatic hydrolysis was conducted in a 2.0 mL centrifugal tube. The standard reaction volume (0.5 mL) consisted 0.2 mol/L PBS (pH 8.0, 425 µL), 10 µg/mL enzyme (0.1 mg/mL, 50 µL), and 10 mmol/L meso-dimethyl ester (200 mmol/mL diester dissolved in DMSO, 25 µL). The mixture was shaken at 30 °C, 1000 rpm for 10 min. Then 1.0 mL of methanol was added to stop the reaction, followed by adding 45 µL of phosphoric acid to acidify the produced monomethyl ester (Wu et al. 2015). After centrifugation at 12,000 rpm for 5 min, the supernatant was filtered through organic filter membrane, and the filtrate was assessed by HPLC. One unit of enzyme activity was defined as the amount of enzyme that released 1 µmol monoester per minute under standard conditions.

The optimal temperature was determined by measuring enzyme activity at pH 8.0 (0.2 mol/L PBS) and different temperatures ranging from 25 °C to 65 °C. The thermostability of the enzyme was evaluated by pre-incubating the purified enzyme (1.0 mg/mL) in 0.2 mol/L PBS (pH 8.0) at 40 °C and 50 °C for up to 3 h. The initial enzyme activity was defined as 100%, and the residual activity was expressed as a percentage of the initial activity. Both the initial and residual enzyme activities were measured using the standard reaction described above at 30 °C in 0.2 mol/L PBS (pH 8.0).

The optimal pH of EstSIT01 was investigated at 30 °C using three different buffers to cover the pH range 6.0 ~ 11.0: 0.2 mol/L phosphate buffer (pH 6.0 ~ 8.0), 0.05 mol/L Tris-HCl (pH 8.0 ~ 10.0), and 0.05 mol/L Gly-NaOH (pH 10.0 ~ 11.0). The pH stability was examined by pre-incubating the purified enzyme (1.0 mg/mL) for 1 h at 30 °C in buffers at different pH values (6.0 ~ 11.0). The initial enzyme activity was defined as 100%, and the residual activity was expressed as a percentage of the initial activity. Both the initial and residual enzyme activities were measured using the standard reaction described above at 30 °C in 0.2 mol/L PBS (pH 8.0).

The effects of inorganic ions (K^+^, Ca^2+^, Mg^2+^, Zn^2+^, Cu^2+^ and NH_4_^+^) on the activity of EstSIT01 were examined at final concentration of 1.0 mmol/L and 5.0 mmol/L at 30 °C and pH 8.0. The enzyme activity determined at standard condition (without adding inorganic ions) was set as control and was defined as 100%. Statistical analysis was performed using the t-test. Here, **** represents *P* < 0.0001, *** represents *P* < 0.001, ** represents *P* < 0.01, and * represents *P* < 0.05.

The values of *K*_m_ and *k*_cat_ were determined at different substrate concentrations ranging from 0.1 mmol/L to 12 mmol/L using diester as the substrate. All other conditions were kept the same as the standard enzymatic assay described above. The *K*_m_ and *k*_cat_ values were calculated using GraphPad Prism software. Experiments were independently performed in triplicate.

### Asymmetric hydrolysis of meso-diester by whole cells of recombinant E. Coli

After induction, cells were harvested by centrifugation at 8000 rpm for 10 min. The sediment was then resuspended in phosphate buffered solution (PBS, 0.2 mol/L, pH 8.0). The whole cell biocatalysis was carried out in 10 mL of 0.2 mol/L PBS (pH 8.0, 100 mL) solution containing 10 g_dcw_/L cells and 100 mmol/L meso-diester at 250 rpm and 30 °C for 24 h. After completion of the reaction, the precipitate was removed by centrifugation. To adjust the pH, concentrated phosphoric acid solution was added to the reaction mixture until the pH reached 2.0. The reaction mixture was then extracted three times with 30 mL of ethyl acetate. The organic phase was combined and dried by anhydrous sodium sulfate. Finally, the product was obtained by removing the organic phase under reduced pressure (Wu et al. 2015). The purity of the product was determined by analyzing its melting point and reverse HPLC, while the *e.e.* value was determined by normal HPLC with chiral column.

### Substrate specificity

The substrate specificity was determined using various *p*-NP esters substrates, including *p*-nitrophenyl acetate (*p*-NPA), *p*-nitrophenyl butyrate (*p*-NPB), *p*-nitrophenyl caproate (*p*-NPC) and *p*-nitrophenyl octanoate (*p*-NPO) (Qingdao Vochem Co., Ltd, China). The release of *p*-nitrophenol was measured at 405 nm using a Multiscan Spectrum spectrophotometer (BioTek, U.S.A) (Jin et al. 2012). The activity of EstSIT01 was examined at 30 °C and pH 8.0 (PBS, 0.2 mol/L). One unit of enzyme activity was defined as the amount of enzyme that released 1 µmol of *p*-nitrophenyl from *p*-NP esters per minute under above mentioned conditions. The relative activity was calculated by considering the maximum enzyme activity as 100%.

### Analysis method

The product formation was quantitatively analyzed using reverse-phase HPLC under the following conditions: A reverse HPLC system (LC-20AT, Shimadzu Co., Ltd, Japan) equipped with a C18 column was employed. The mobile phase consisted of methanol/pH 2.5 water (65:35, v/v) with a flow rate of 1.0 mL/min at 20 °C. Detection was performed at a UV wavelength of 210 nm. The retention time for the dimethyl ester and monomethyl ester were 7.5 min and 10.5 min, respectively. To construct the standard curve for monomethyl ester, the same analysis method was followed. For chiral analysis of the *e.e.* value of the monomethyl ester, the sample was analyzed using HPLC (LC-20AT, Shimadzu Co., Japan) equipped with a chiral column (Chiralcel OJ-H, Daicel Co., Ltd, Japan). The mobile phase consisted of n-hexane, isopropanol, and trifluoroacetic acid (92:7.8:0.2, v/v) with a flow rate of 0.5 mL/min at 20 °C. Detection was performed at a wavelength of 220 nm (Wei et al. 2013). The retention time for (4*R*, 5*S*)-monomethyl ester and (4*S*, 5*R*)-monomethyl ester were 36.9 min and 41.6 min, respectively.

### Docking and MD simulations

The modeling was performed using Robetta (https://robetta.bakerlab.org/ submit.php) with RoseTTAFold (Baek et al. 2021). The resulting 3D model was validated with the SAVES v6.0 server (https://saves.mbi.ucla.edu/) for model quality estimation. Docking of meso-diester into the active site region was performed using Webina, which is a web assembly library that runs AutoDock Vina entirely in a web browser (Kochnev et al. 2020; Morris et al. 2009). The size of docking box was set to 16Å×16Å×16Å (x, y, and z axis), and the center coordinates of the docking box were specified as (-10, -10, -17). The protein (EstSIT01) was prepared by adding explicit hydrogen atoms using the H + + software (http://newbiophysics.cs.vt.edu/H++/ index.php). ACPYPE was used to construct the force field parameters of the substrates (meso-diester) (Sousa da Silva and Vranken 2012). The system was solvated with TIP3P water molecules using a 12.0 Å solvent buffer between the solute and the closest edge of the unit cell. The Amber ff14SB force field was applied to the protein and substrate. Amber 12 was used for performing minimization, heating, and equilibration (Gotz et al. 2012), and the GPU-accelerated Gromacs v2020.03 was used to perform final molecular dynamics. The protein structures were visualized using the PyMOL program (The PyMOL Molecular Graphics System, Version 2.0 Schrödinger, LLC.).

## Results and discussion

### Discovery the target esterase based on genome mining and activity test

**Fig. 1:**
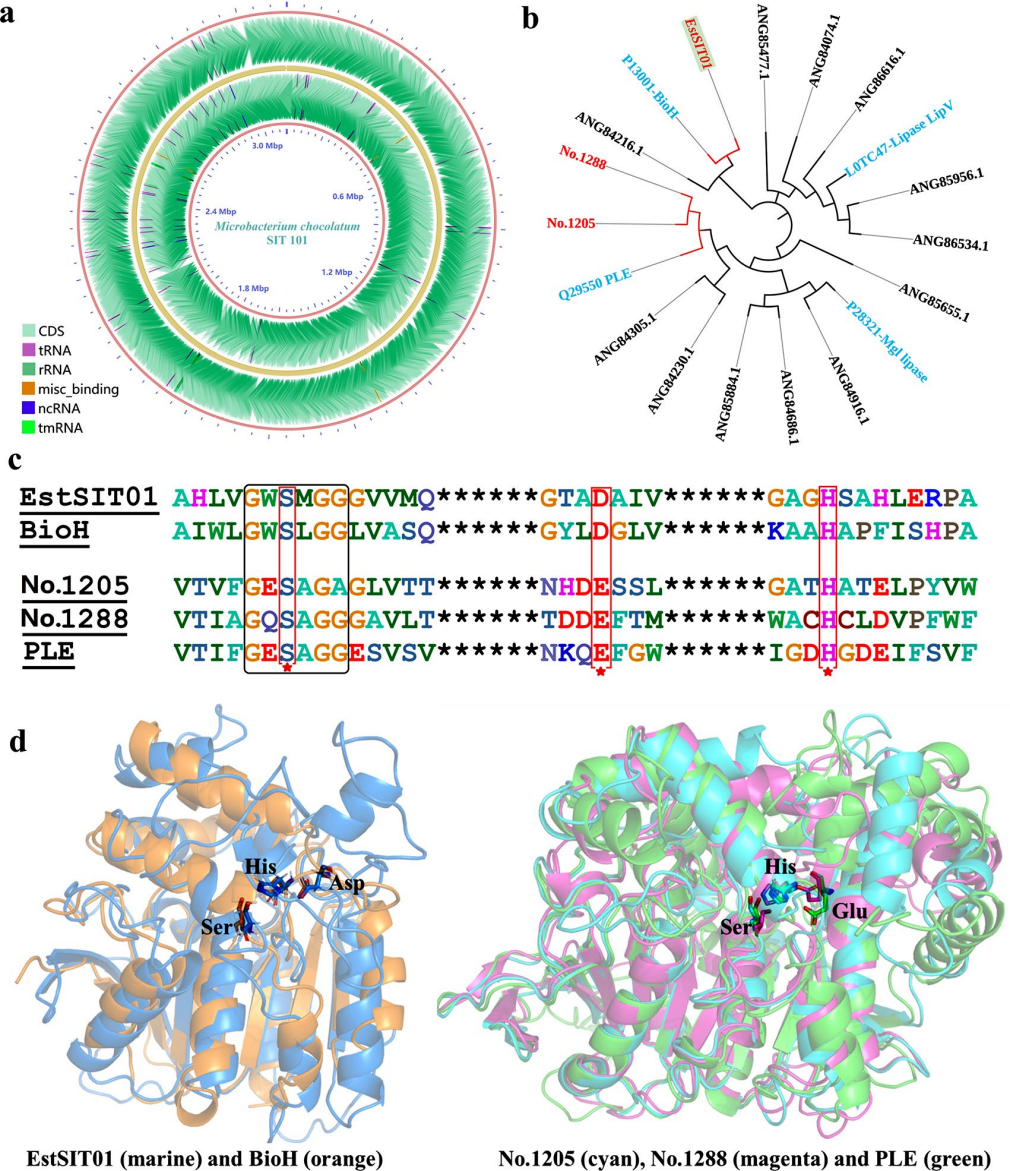
Sequence and structure analysis. (**a**) Genomic map of *Microbacterium chocolatum* SIT101. (**b**) Phylogenetic tree of four reported enzymes (cyan) and 15 enzymes in this article. (**c**) Multiple sequence alignment of the partial sequences between three potential target enzymes (EstSIT01, No.1205 and No.1288) and two reported stereoselective esterases, the catalytic triad was marked by the red five-pointed stars. (**d**) Modeling structure match

In our previous study, we demonstrated that both the whole cell and the cell-free extract of *Microbacterium chocolatum* SIT101 exhibited remarkable catalytic activity in the asymmetric hydrolysis of meso-dimethyl ester, yielding (4*S*, 5*R*)-monomethyl ester with excellent stereoselectivity. These encouraging findings motivated us to identify the gene sequence of the target esterase from *Microbacterium chocolatum* SIT101 and explore its heterologous expression in *E. coli*. However, since no genome sequence of *Microbacterium chocolatum* was available in the public database, we sequenced the complete genome of SIT101 and submitted it to the NCBI (Accession number: CP015810.1-CP015813.1). According to the final assembly results (Fig. 1a), the size of the genome was 3,168,630 bp and comprised one chromosome and three plasmids, with a GC content of 69.99%. Annotation of the genome revealed the presence of 3,024 CDS genes, 6 rRNA operons, 49 tRNA genes and 82 pseudogenes. Through genome sequence annotation and manual inspection, we identified 15 ester hydrolase genes, including α/β hydrolases and esterases, after removing duplications and truncated sequences (Additional file 1: Table S1).

To investigate the relationship between these ester hydrolases and known lipolytic enzymes, we constructed a phylogenetic tree using the obtained sequences and four known lipolytic enzymes (Fig. 1b). Interestingly, ANG84115.1 (EstSIT01) and BioH from *Escherichia coli* were clustered together in the same branch. BioH is an essential pimeloyl-ACP methyl ester cleavage enzyme involved in biotin biosynthesis and has been identified as an organic solvent-tolerant esterase with favorable stereoselectivity in the production of enantiomerically pure chemicals (Bo et al. 2010; Hang et al. 2019). Moreover, ANG84283.1 (No.1288) and ANG84352.1 (No.1205) exhibited sequence similarity to PLE (Pig liver esterase), which has the ability to selectively hydrolyze meso-diester stereoselectively for synthesis of (4*S*,5*R*)-monoester (*e.e.* value = 91%) (Chen et al. 2005). Multiple sequence alignment further revealed that EstSIT01, No.1205 and No.1288 possess the conserved GXSXG motif and the catalytic triad (serine, histidine and aspartic acid/glutamic acid) in the active center (Fig. 1c and d), indicating their potential for ester hydrolysis based on sequence and structural analysis. Consequently, No.1205, No.1288 and EstSIT01 were cloned into *E. coli* BL21 (DE3) for subsequent activity verification (Additional file 1: Table S2&Fig. S1).

The recombinant plasmids containing No.1205, No.1288, and EstSIT01 were introduced into *E. coli* BL21 (DE3) cells, which were then cultivated in LB medium supplemented with 100 µg/mL ampicillin at 200 rpm and 37 °C. Once the optical density at 600 nm (OD_600_) reached 0.6–0.8, protein overexpression was induced by adding 0.2 mmol/L IPTG. After 8 h of induction at 200 rpm and 30 °C, the cells were harvested and directly evaluated for their ability to hydrolyze meso-dimethyl ester. Interestingly, No.1205 and No.1288 did not exhibit any activity towards meso-dimethyl ester. While the recombinant *E. coli* expressing EstSIT01 demonstrated significantly enhanced activity compared to that of *Microbacterium chocolatum* SIT101.

Identifying enzymes with precise functionalities from the genome poses particular complexities. Through the construction of phylogenetic trees, sequence and structure analysis, and a small amount of experimental validation, we ultimately identified EstSIT01, which can be utilized for the synthesis of (4*S*, 5*R*)-monomethyl ester. Previous studies have also reported cases of discovering enzymes with specific functions using similar methods (Meinert et al. 2021). Adopting this technological approach, we could narrow down the search and screen a small number of candidate enzymes. However, achieving 100% accurate prediction is not feasible, further activity experiments are required for verification.

The specific activity of recombinant *E. coli* whole cell was measured to be 243 U/g_cdw_, which was approximately 40 times higher than that of *Microbacterium chocolatum* SIT101. Additionally, the (4*S*, 5*R*)-monoester can be synthesized in phosphate buffered solution without any organic solvent. This remarkable increase in activity underscores the potential of EstSIT01 for efficient enzymatic hydrolysis reactions, making it a promising candidate for various industrial applications.

### Asymmetric hydrolysis of meso-diester by EstSIT01

The HPLC standard curve of (4*S*, 5*R*)-monoester was first established to facilitate the determination of enzymatic activity (Additional file 1: Fig. S2a). Upon completion of the reaction, (4*S*, 5*R*)-monoester was obtained with an impressive 99% analytical yield (Additional file 1: Fig. S2b&2c) and *e.e.* value (Additional file 1: Fig. S2d&2e). Following separation and purification, the pure product exhibited a melting point of 153–155 °C, which was consistent with the reported value of (4*S*, 5*R*)-monoester. Carboxylesterases represent a class of hydrolases, characterized by their high catalytic efficiency. These enzymes are recognized for their ability to hydrolyze carboxylic esters. It has been reported that carboxylesterases, particularly Pig Liver Esterase (PLE), exhibit remarkable enantioselectivity and stereoselectivity, rendering them extensively utilized in biosynthesis (Zhou et al. 2019). However, when preparing (4*S*, 5*R*)-monoester, the *e.e.* value and yield of PLE were 91% and 90%, respectively (Chen et al. 2005), which is inferior to the 99% achieved by EstSIT01. At present, no other enzymes have been observed to outperform this enzyme in terms of production of (4*S*, 5*R*)-monoester.

Based on the preceding results, it is unequivocally evident that EstSIT01 harbors both novelty and economic utility. Firstly, EstSIT01 demonstrates high heterologous expression levels in *Escherichia coli*, rendering it relatively inexpensive to obtain. Secondly, the enzyme exhibits high conversion and stereoselectivity, both reaching up to 99%, thus substantially decreasing downstream processing costs. Additionally, the reaction can be conducted in a pure aqueous solution without requiring any organic cosolvents, making the process environmentally friendly. Moreover, immobilizing the enzyme or whole cell can further reduce expenses. In summary, employing EstSIT01 for biotin intermediates preparation presents a promising cost-effective industrial pathway.

### *Purification and characterization of* EstSIT01

Following inducible expression, the harvested cells were disrupted using ultrasonication, and the resulting supernatant was obtained through centrifugation. The enzyme was subsequently eluted by 250 mmol/L imidazole buffer and concentrated by ultrafiltration. The purified protein exhibited a concentration of 3.56 mg/mL, accounting for 28.2% of the total soluble protein in the cell. In the SDS-PAGE analysis (Additional file 1: Fig. S2f), a clear and distinct band appeared at around 40 kDa, aligning closely with the expected theoretical molecular weight of 39.35 kDa. Notably, this observed molecular weight bears a striking resemblance to that of EstZ3, which is approximately 39 kDa (Bayer et al. 2010).

**Fig. 2:**
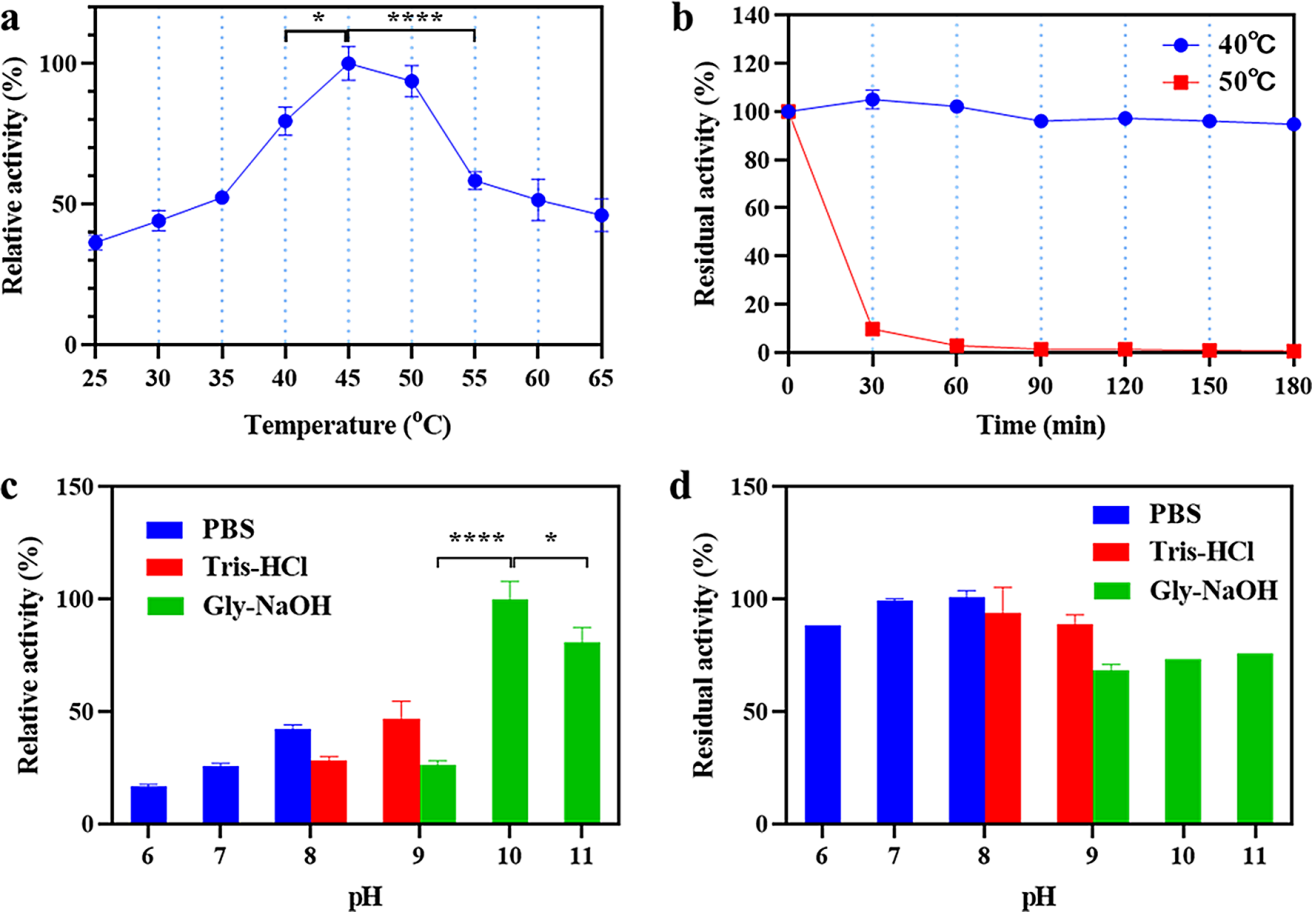
Enzymatic property of EstSIT01. (**a**) Effect of temperature on enzyme activity of EstSIT01. The maximum activity of EstSIT01 was defined as 100%. (**b**) The thermostability of EstSIT01 at different temperature. The initial enzyme activity was defined as 100%. (**c**) Effect of pH on activity of EstSIT01. The maximum enzyme activity was defined as 100%. (**d**) Effect of pH on the stability of EstSIT01. The initial enzyme activity was defined as 100%

To investigate the enzymatic properties of EstSIT01, its activity and stability under different pH and temperature conditions were characterized (Fig. 2). The results revealed that EstSIT01 displayed optimal activity at 45 °C, and the activity at 50 °C did not decrease significantly. However, it sharply declined to 58% of its activity at 45 °C when the temperature reached 55 °C, and it retained only 46% of its maximum activity even at 65 °C (Fig. 2a). Furthermore, the enzyme exhibited no significant loss of activity after incubation at 40 °C for 3 h. Nonetheless, its stability was compromised at 50 °C, as evidenced by Fig. 2b, where the residual activity dropped to just 10% after a 1-hour incubation period. The optimal temperature for EstSIT01 closely aligns with that of esterase E53 (40 °C) (Ding et al. 2021). Both of these enzymes belong to the mesophilic enzyme category, and temperatures exceeding 45 °C may potentially disrupt their tertiary structure. It is widely acknowledged that enzymes with strong thermal stability offer various advantages in industrial applications, particularly in terms of transportation, storage, and long-term catalytic reactions. However, EstSIT01 exhibits moderate thermal stability at 50 °C. The moderate thermal stability is presumably rooted in its microbial source, *Microbacterium chocolatum*, which proliferates optimally in a normal temperature environment approximating 28 °C and does not possess thermophilic traits. To enhance its industrial applicability potential, protein engineering may be imperative for further augmenting its stability.

EstSIT01 demonstrated the highest activity at pH 10.0 in a Gly-NaOH buffer (Fig. 2c), and the activity exhibited a declining trend as the pH value exceeded 10.0. Only a few esterases exhibit optimal activity at pH values exceeding 9, yet EstSIT01 shows its optimal activity at pH 10, indicating that EstSIT01 is an alkaline-active esterase (Rong et al. 2018). Notably, a decrease in activity to 16% was observed when the pH was lowered to 6.0, likely attributed to the protonation of the side chain of His330 in the catalytic triad (Fu et al. 2013). The impact of pH on enzyme stability were also evaluated. It was observed that no significant decrease in enzyme activity occurred following a 1-hour incubation at pH 7.0 and 8.0 at 30 °C. However, a modest 10% decrease in activity was noted at pH 6.0 within the same timeframe. Notably, EstSIT01 retained less than 70% of its initial activity at pH 9.0, 10.0, and 11.0 in the Gly-NaOH buffer after 1 h (Fig. 2d). These findings indicate that EstSIT01 displays robust stability under neutral to slightly alkaline pH conditions. Excessively high or low pH levels can compromise stability and activity. Thus, maintaining a pH close to 8.0 in the reaction mixture is essential for efficient biotransformation, especially when dealing with high substrate loads. EstSIT01 follows a trend similar to other microbial esterases, exhibiting increased activity in slightly alkaline environments, as seen in the case of the esterase AcEst1 (pH 9.0) (Dou et al. 2020). EstSIT01 possesses relatively high activity in alkaline conditions, making it a promising candidate for applications in industries such as detergents.

**Fig. 3:**
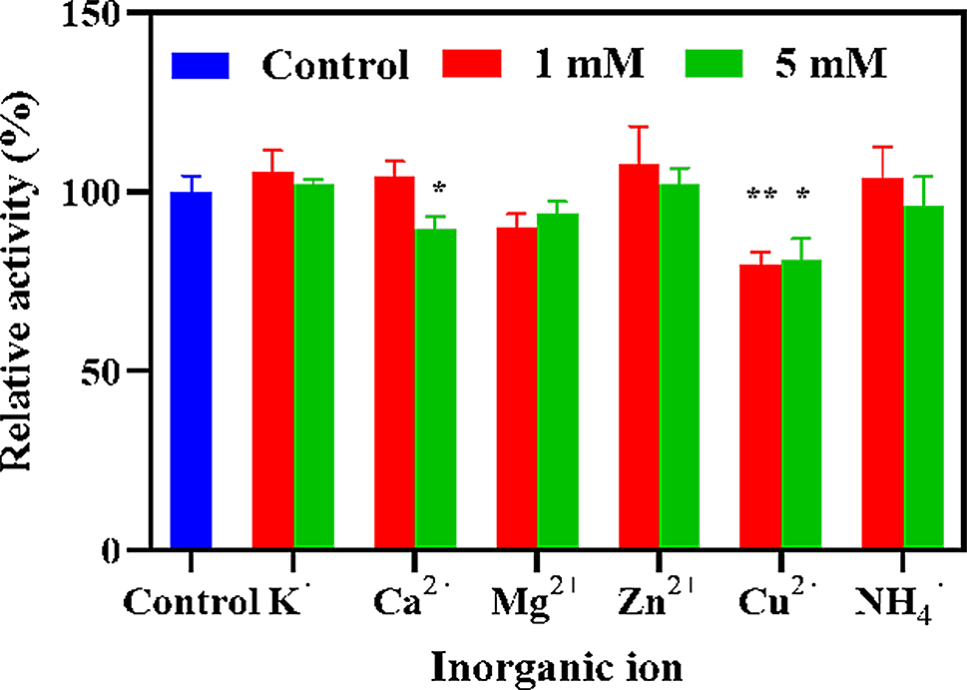
Effect of various inorganic ions on the activity of EstSIT01. The enzyme activity was determined at 30^o^C in 0.2 mol/L PBS (pH 8.0). The blue column represents none inorganic ions; the red and green column represents 1.0 mmol/L and 5.0 mmol/L inorganic ions, respectively

The influence of various inorganic ions on the activity of EstSIT01 was investigated, and the results were shown in Fig. 3. Most of the tested inorganic ions did not have a significant impact on the enzyme activity, except for Ca^2+^ and Cu^2+^ (Fig. 3). In the presence of 5.0 mmol/L Ca^2+^, the activity of EstSIT01 was notably decreased by approximately 10% compared to the control group. Similarly, Cu^2+^ at concentrations of 1.0 mmol/L and 5.0 mmol/L caused a reduction in activity of approximately 20%. These findings provide evidence that Ca^2+^ and Cu^2+^ exert a moderate inhibitory influence on EstSIT01’s activity. Likewise, both EstATII and AcEst1 exhibit significant inhibition in the presence of Cu^2+^ (Dou et al. 2020; Mohamed et al. 2013), similarly, E53’s activity is impeded by Ca^2+^ (Ding et al. 2021). However, Ca^2+^ appears to have a potential enhancing effect on the activity of est3S (Lee et al. 2021).

To determine the kinetic parameters of EstSIT01 towards diester, various concentrations of diester ranging from 0.1 mmol/L to 12 mmol/L were used. The Michaelis-Menten plot was generated using GraphPad Prism (Additional file 1: Fig. S2g). The kinetic analysis revealed that the *K*_m_ value, which represents the substrate concentration at which the enzyme reaches half of its maximum velocity, was determined to be 0.147 mmol/L. The *k*_*cat*_ value, denoting the number of substrate molecules EstSIT01 can convert to product per unit time, was calculated to be 5.808 *s*^− 1^. These kinetic parameters provide valuable insights into the catalytic efficiency and substrate binding affinity of EstSIT01 towards diester. These kinetic parameters offer valuable insights into EstSIT01’s catalytic efficiency and its affinity for diester substrates. Importantly, we did not observe any inhibition at high substrate concentrations, which is a crucial consideration for industrial production.

### Substrate specificity of esterase EstSIT01

**Fig. 4:**
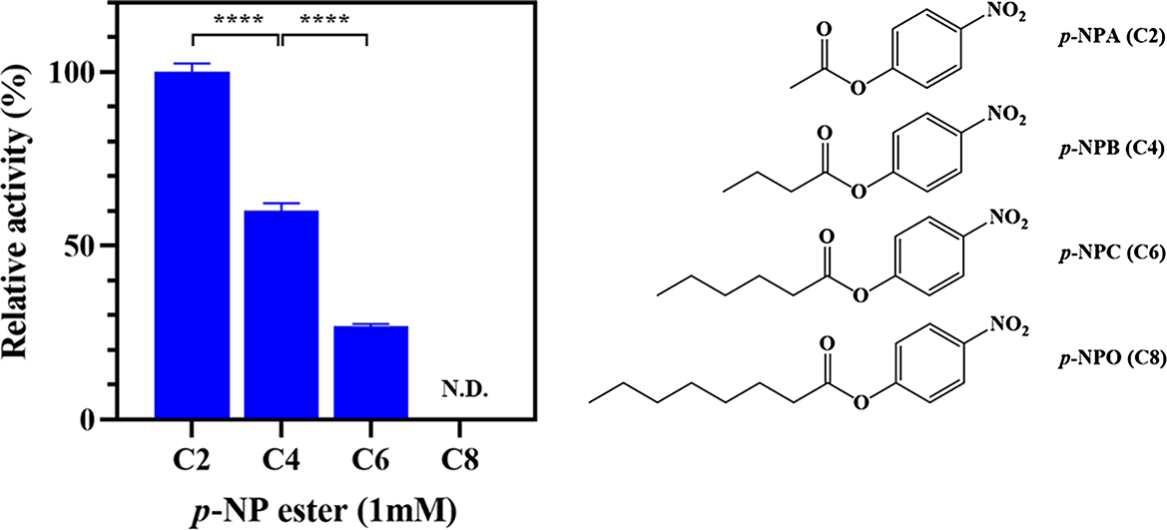
Substrate spectrum of EstSIT01 towards various *p*-NP esters. *p*-NP esters of various lengths were assayed at 30 °C in 0.2 mol/L phosphate buffer solution (pH 8.0). The activity for *p*-nitrophenyl acetate (0.98 U/mg) was defined as 100%

To investigate the enzyme specificity of EstSIT01 towards different acyl-chain lengths in the substrate, *p*-nitrophenyl esters (*p*-NP esters) were used as substrates. The substrate spectrum analysis revealed that EstSIT01 displayed a preference for short-chain *p*-NP esters (Fig. 4). Among the tested substrates, EstSIT01 exhibited the highest activity towards *p*-nitrophenyl acetate (*p*-NPA) with an activity of 0.98 U/mg. The hydrolysis of *p*-nitrophenyl butyrate (*p*-NPB) was approximately 60% of the activity observed for *p*-NPA, while the activity further decreased to 27% for *p*-nitrophenyl caproate (*p*-NPC) with a longer acyl-chain length (C6). No activity was detected for *p*-nitrophenyl octanoate (*p*-NPO), which has an acyl-chain length of C8. It’s worth noting that, as reported, lipases typically exhibit a preference for substrates with relatively longer alkyl chains (> C6) (Matinja et al. 2022; Ng et al. 2021), whereas esterases are known for their ability to hydrolyze esters with short-chain (≤ C8) acyl groups (Barzkar et al. 2021). Therefore, EstSIT01 should be categorized as an esterase rather than a lipase.

### Phylogenetic and catalytic mechanism analysis of EstSIT01

**Fig. 5:**
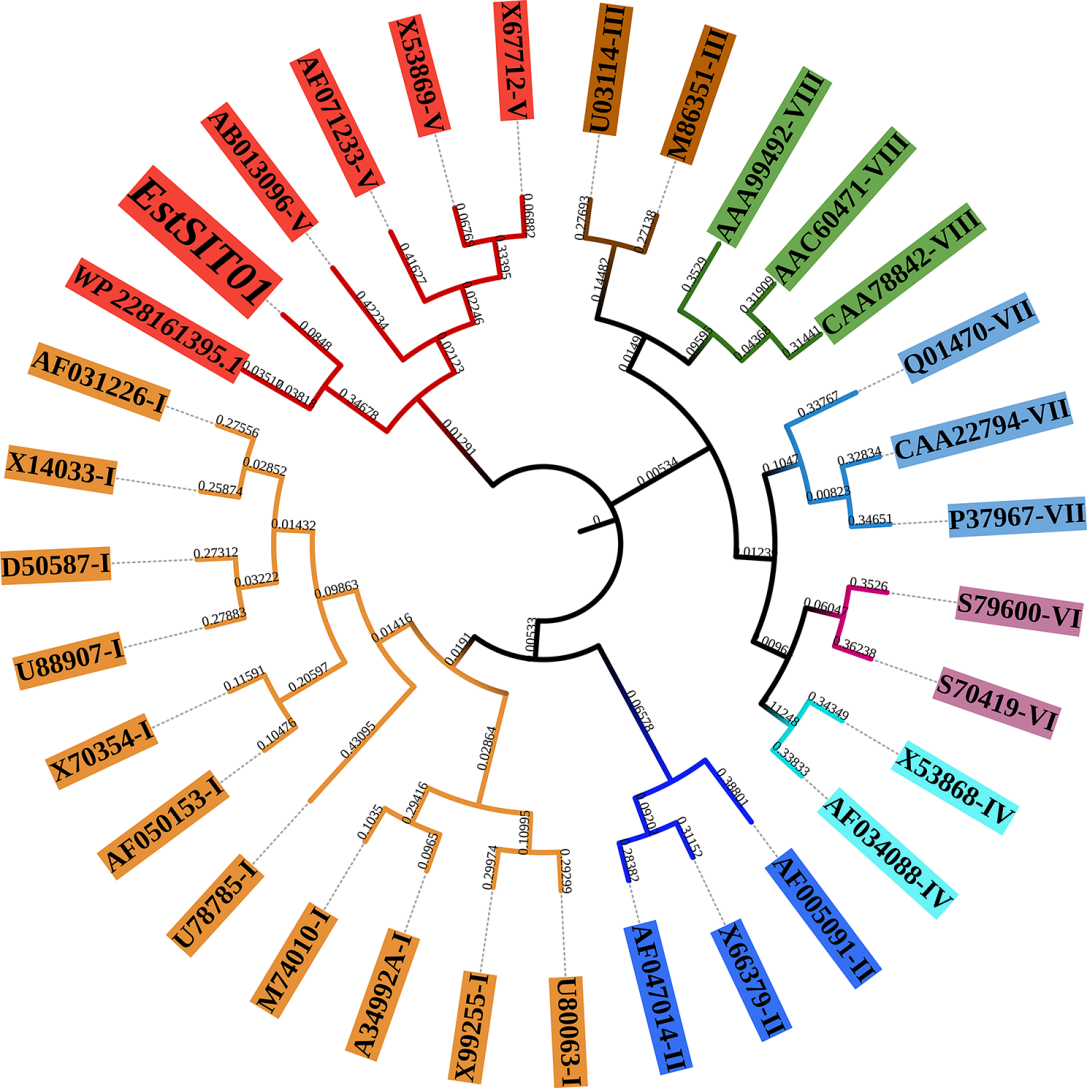
Phylogenetic analysis of EstSIT01 and WP_228161395.1 from *Microbacterium testaceum*. The classical classification Family I-VIII of bacterial esterases and lipases based mainly on the similarity of amino acid sequences and some fundamental biological properties (Arpigny et al. 1999)

The DNA sequence analysis of EstSIT01 revealed that it consists of a 1110-bp open reading frame (ORF) encoding a protein with 370 amino acids. The amino acids sequence of EstSIT01 differs significantly from currently reported esterase sequences. A blast search against the UniProtKB reviewed database revealed that it exhibits a relatively high degree of similarity to the functionally verified BioH (UniProtKB ID: P13001), but with only 35.2% identity. A blast search against the NCBI Non-redundant protein database (Nr) was also conducted, the results indicate that EstSIT01 has a high sequence similarity of 84.32% with an α/β hydrolase from *Microbacterium testaceum* (Accession ID: WP_228161395.1). However, this enzyme is merely a hypothetical and automatically predicted one in the database, and has not yet been characterized. To the best of our knowledge, it was the first report on an esterase from *Microbacterium* sp. for its discovery and characterization. Phylogenetic analysis (Fig. 5) was conducted to further investigate the relationship of EstSIT01 with other related proteins. The analysis revealed that both EstSIT01 and WP_228161395.1 exhibited a high sequence similarity with carboxylesterases belonging to family V. This suggests that EstSIT01 should be classified into the family V. Esterases in this family shared sequence similarity of 20–25% with other enzymes such as epoxide hydrolases, dehalogenases, haloperoxidases, and various bacterial non-lipolytic enzymes, these enzymes possess the typical α/β-hydrolase fold and a catalytic triad, which are characteristic features of this family (Arpigny et al. 1999). This classification provides insights into the potential function and structural features of EstSIT01, expanding our understanding of α/β hydrolases in *Microbacteria*.

**Fig. 6:**
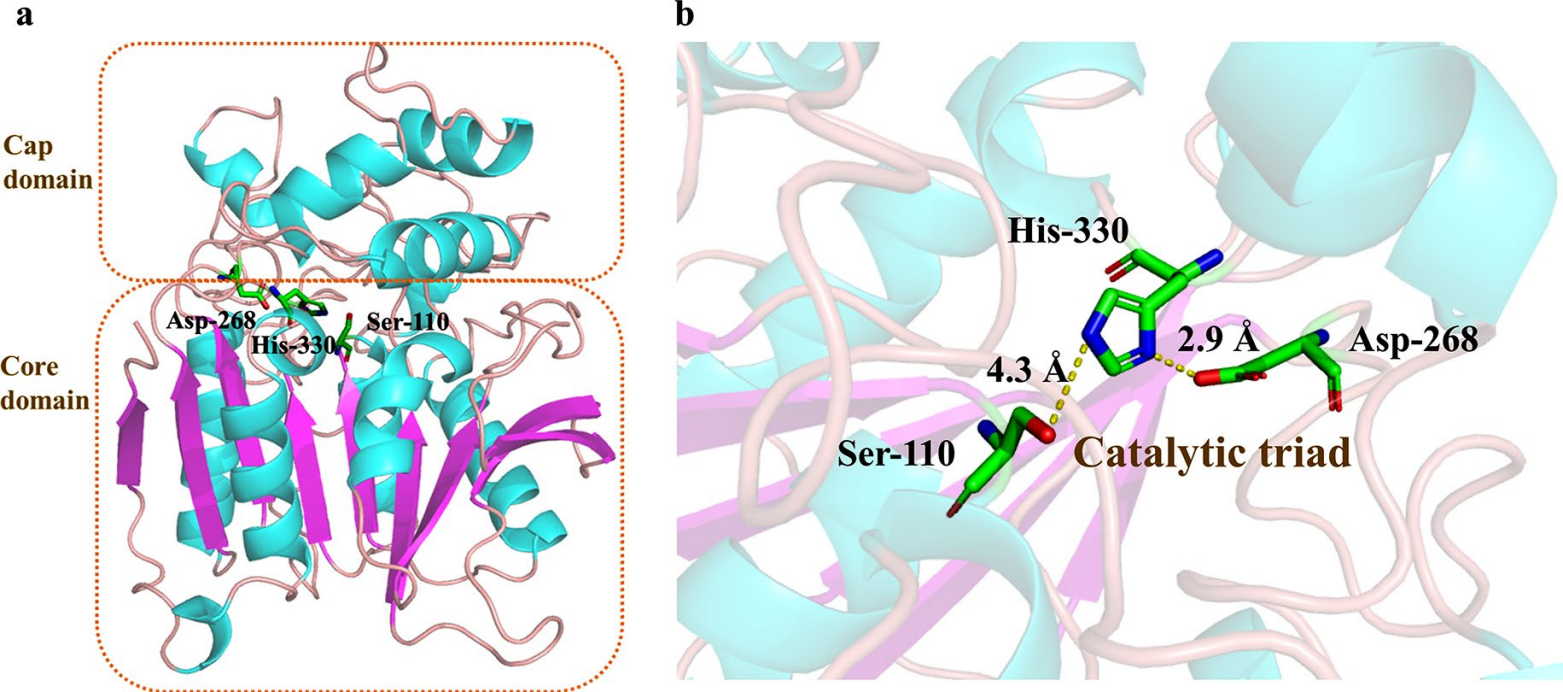
The structure of EstSIT01. (**a**) The modeling structure of EstSIT01. (**b**) The catalytic triad (Ser110-His330-Asp268).

The modeling structure of EstSIT01 was evaluated using SAVES v6.0, and several evaluation tools were employed, including Errat (Colovos and Yeates 1993), Verify 3D (Bowie et al. 1991), and PROCHECK (Laskowski et al. 1993). The overall quality factor was found to be 95.53 (Additional file 1: Fig. S3a), indicating a high-quality structure. Approximately 90.27% of the residues had an averaged 3D-1D score greater than or equal to 0.1 (Additional file 1: Fig. S3b), indicating good agreement between the predicted 3D structure and the corresponding 1D amino acid sequence. Only 0.7% of the residues (Asp5 and Asp27) were located in the disallowed region, suggesting overall reliability of the model (Additional file 1: Fig. S3c). The overall structure of EstSIT01 (Fig. 6a and b) revealed the presence of a typical α/β-hydrolase fold catalytic domain (core domain) and a cap domain located above the core domain. The core domain contained the catalytic triad (Ser110-His330-Asp268) necessary for enzymatic activity, while the cap domain was composed of five α-helices connected by loops. The α/β hydrolase core domain consisted of nine central β-sheets surrounded by six α-helices, with a long loop followed by a short β-sheet attached to the C-terminal.

To understand the binding mode of EstSIT01 with the substrate meso-diester, molecular docking was performed. Interestingly, no docking conformation for the formation of (4*R*, 5*S*)-monoester (the undesired product) was generated, indicating that the configuration of meso-diester binding with EstSIT01 for formation (4*R*, 5*S*)-monoester may not be energetically favorable. The best docking configuration producing (4*S*, 5*R*)-monoester (the desired product) with a binding energy of -5.1 kcal/mol.

**Fig. 7:**
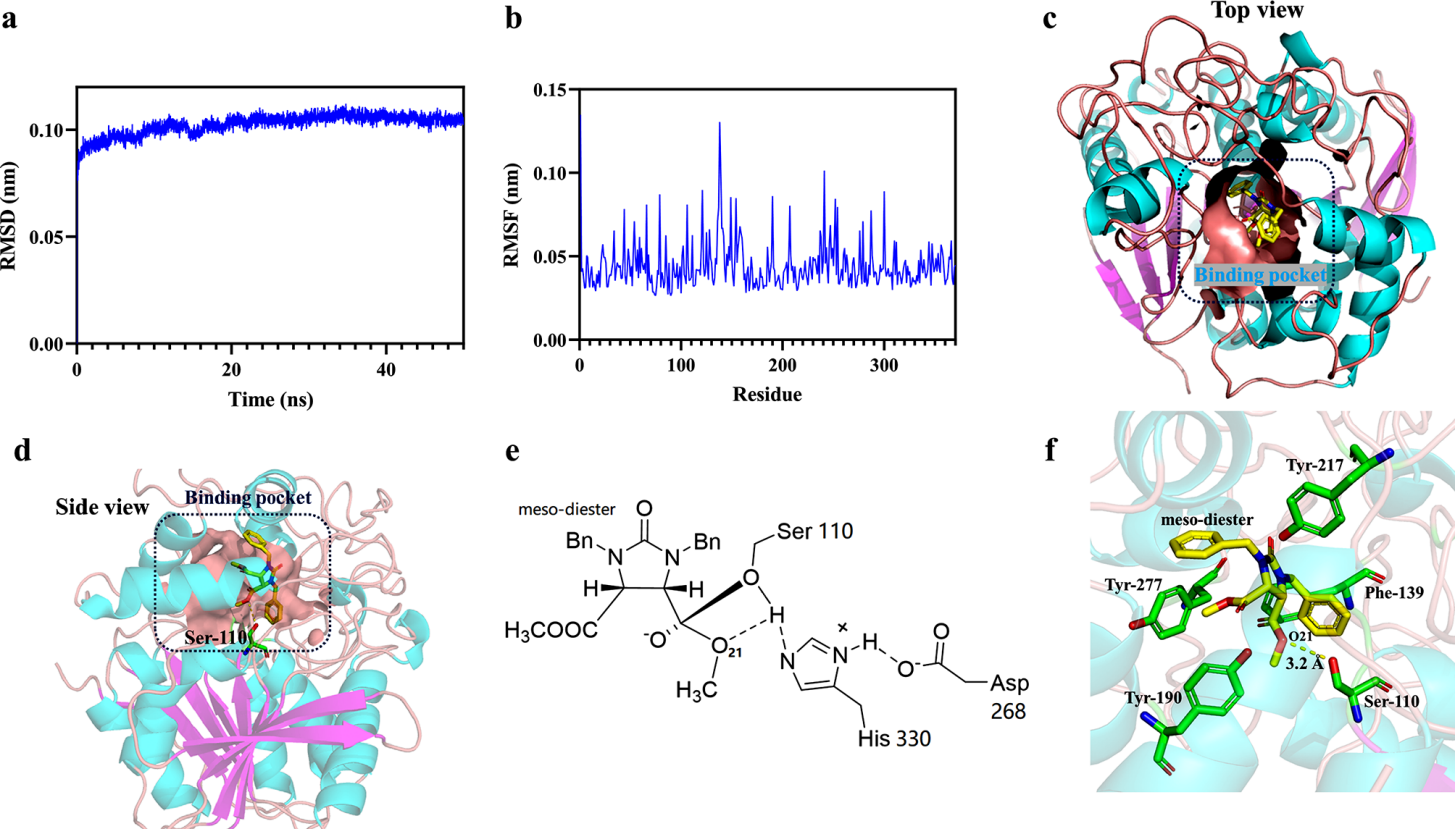
Molecular dynamics simulations of EstSIT01-diester. (**a**) 50 ns RMSD plot. (**b**) RMSF plot of last 10 ns MD simulations. (**c**) Top view of ligand binding pocket (yellow stick represented meso-diester). (**d**) Side view of ligand binding pocket. (**e**) The tetrahedral transition state of EstSIT01-diester. (**f**) Hydrophobic residues around the substrate

In order to assess the stability of the EstSIT01-diester complex, molecular dynamics simulations were conducted for 50 ns in aqueous solution. The root mean square deviation (RMSD) plot for the backbone atoms of the EstSIT01-diester complex reached equilibrium rapidly and remained around 0.11 nm, indicating the stability of the complex (Fig. 7a). During the last 10 ns of the molecular dynamics simulations, the root mean square fluctuation (RMSF) analysis showed that the residues Val79, Leu121, Gly138, Phe139, Tyr190, Met241, and Gln300 exhibited higher flexibility, with fluctuations ranging from 0.09 nm to 0.13 nm (Fig. 7b). In contrast, the catalytic triad residues (Ser110, Asp268, and His330) showed very low fluctuations, indicating their stability and important role in catalysis. It was reported that rigidifying flexible loops at appropriate sites may enhance the stability of the protein (Rahban et al. 2022), consequently, the highly flexible sites mentioned above can serve as potential mutation sites for enhancing protein stability.

To investigate the reasons for the stereoselectivity of EstSIT01, a snapshot was taken from the 50 ns MD simulation trajectory. The conformation of EstSIT01-diester revealed that the substrate meso-diester spontaneously turned sideways to pass through the narrow entrance at the top of the cap domain and entered the hydrophobic pocket (Fig. 7c). The presence of a tunnel from the protein surface to the binding site (Ser110) was also identified and visualized using the Caver 3.02 plugin in PyMol (Additional file 1: Fig. S3d). Once the substrate entered the binding pocket, it approached the active center, where the Ser110 residue formed a hydrogen bond with the O21 of the substrate at a distance of 3.2 Å (Fig. 7d). The catalytic mechanism involved the deprotonation of the hydroxyl hydrogen of Ser110 by His330, followed by the nucleophilic attack of the Ser110 oxygen on the carbonyl carbon of the meso-diester, leading to the formation of a tetrahedral transition state (Fig. 7e). This state was quickly corrupted through proton transfer with the hydrogen of His330, resulting in the release of methanol and the formation of the acyl-enzyme complex (Dou et al. 2022). Furthermore, several aromatic amino acids, including Phe139, Tyr190, Tyr217, and Tyr277, interacted with the ligand through hydrophobic interactions (Fig. 7f). Among these, Phe139 and Tyr190 exhibited relatively higher flexibility during the last 10 ns of the simulations. These hydrophobic side chains contributed to the formation of a hydrophobic pocket that accommodated the meso-diester substrate, which is critical for its enantioselectivity. Based on the analysis of MD simulations, it can be concluded that the enantioselectivity of EstSIT01 toward meso-diester is attributed to the narrow entrance and unique structure of the binding pocket, along with the interactions of specific residues and the catalytic triad involved in the catalytic mechanism.

## Conclusions

In this study, we rapidly identified EstSIT01, a novel carboxylesterase from *Microbacterium chocolatum* SIT101, through genome mining and phylogenetic analysis. EstSIT01 serves as an efficient biocatalyst, enabling stereoselective meso-diester hydrolysis, yielding (4*S*, 5*R*)-monoester in high yield and enantiomeric excess, which is an essential intermediate for *d*-biotin synthesis. Optimal conditions for EstSIT01 are pH 10.0 and 45 ^o^C, with *K*_m_ and *k*_cat_ values of 0.147 mmol/L and 5.808 s^− 1^, respectively. EstSIT01 displays a preference for short-chain *p*-NP esters. Its high activity, stereoselectivity, and favorable kinetics position EstSIT01 as a promising candidate for synthesizing valuable carboxylic acids and esters. Our work also provides valuable references for the rapid discovery of other new enzymes from the natural sources.

### Electronic supplementary material

Below is the link to the electronic supplementary material.

Supplementary Material 1

Supplementary Material 2

## Data Availability

All data generated or analyzed during this study are included in this published article (and its additional files).
