# Supplementary figures and images for: Discovery, characterization and mechanism of a Microbacterium esterase for key d-biotin chiral intermediate synthesis
Source: Bioresour Bioprocess. 2024 Jun 16;11(1):59. doi: 10.1186/s40643-024-00776-2 (PMC11180644; doi:10.1186/s40643-024-00776-2)

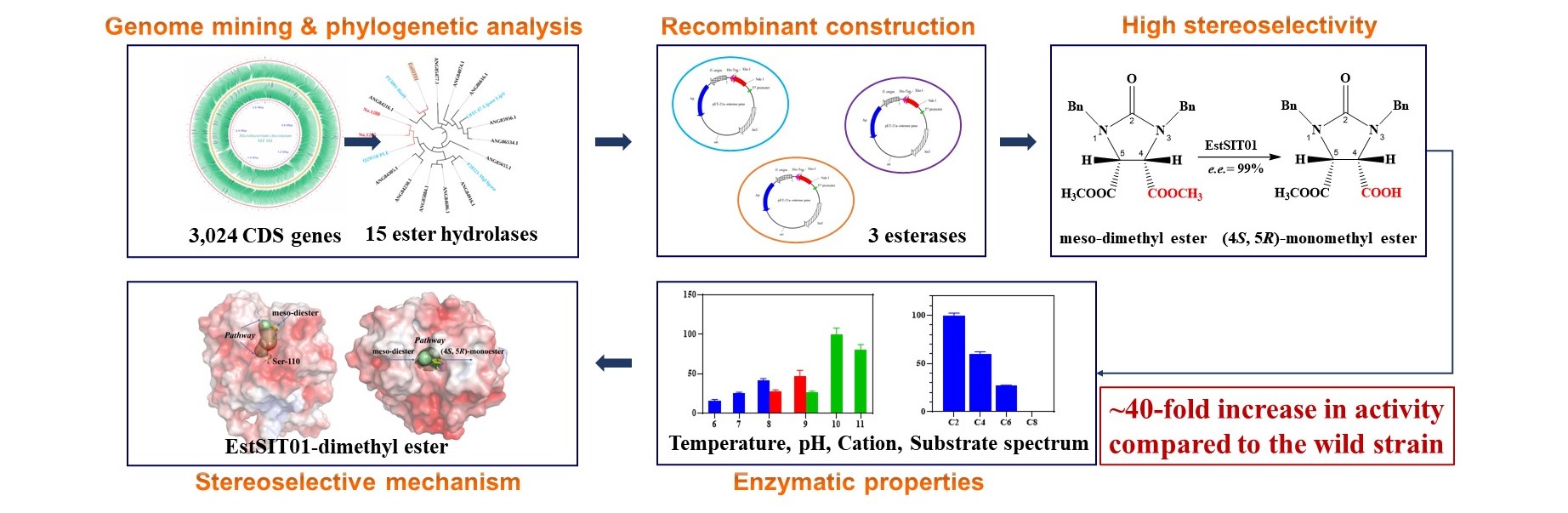

Supplement: Supplementary file 1 — Supplementary Material 1 [file 40643_2024_776_MOESM1_ESM.jpg]
