# Supplementary material for: Discovery, characterization and mechanism of a Microbacterium esterase for key d-biotin chiral intermediate synthesis
Source: Bioresour Bioprocess. 2024 Jun 16;11(1):59. doi: 10.1186/s40643-024-00776-2 (PMC11180644; doi:10.1186/s40643-024-00776-2)
Supplement: Supplementary file 2 — Supplementary Material 2 [file 40643_2024_776_MOESM2_ESM.docx]

Submitted to: ***Bioresources and Bioprocessing***

*Supporting information*

**Discovery, characterization and stereoselective mechanism of a novel esterase from *Microbacterium chocolatum* SIT101 for efficient synthesis of key chiral intermediate of *d*-biotin**

Xinjia Li ^1,2^, Haoran Yu ^3^, Shengli Liu ^4^, Baodi Ma ^1^, Xiaomei Wu ^1^, Xuesong Zheng ^5^, Yi Xu ^1*^

^1^ School of Chemical and Environmental Engineering, Shanghai Institute of Technology, 100 Haiquan Road, Shanghai 201418, China

^2^ Xianghu Laboratory, Hangzhou 311231, China

^3^ Institute of Bioengineering, College of Chemical and Biological Engineering, Zhejiang University, Hangzhou 310027, Zhejiang, China

^4^ Shandong Lonct Enzymes Co., Ltd., Linyi 276400, China

^5^ School of Perfume and Aroma Technology, Shanghai Institute of Technology, 100 Haiquan Road, Shanghai 201418, China

*Correspondence and requests for materials should be addressed to Yi Xu (email: xuyi@sit.edu.cn)

**
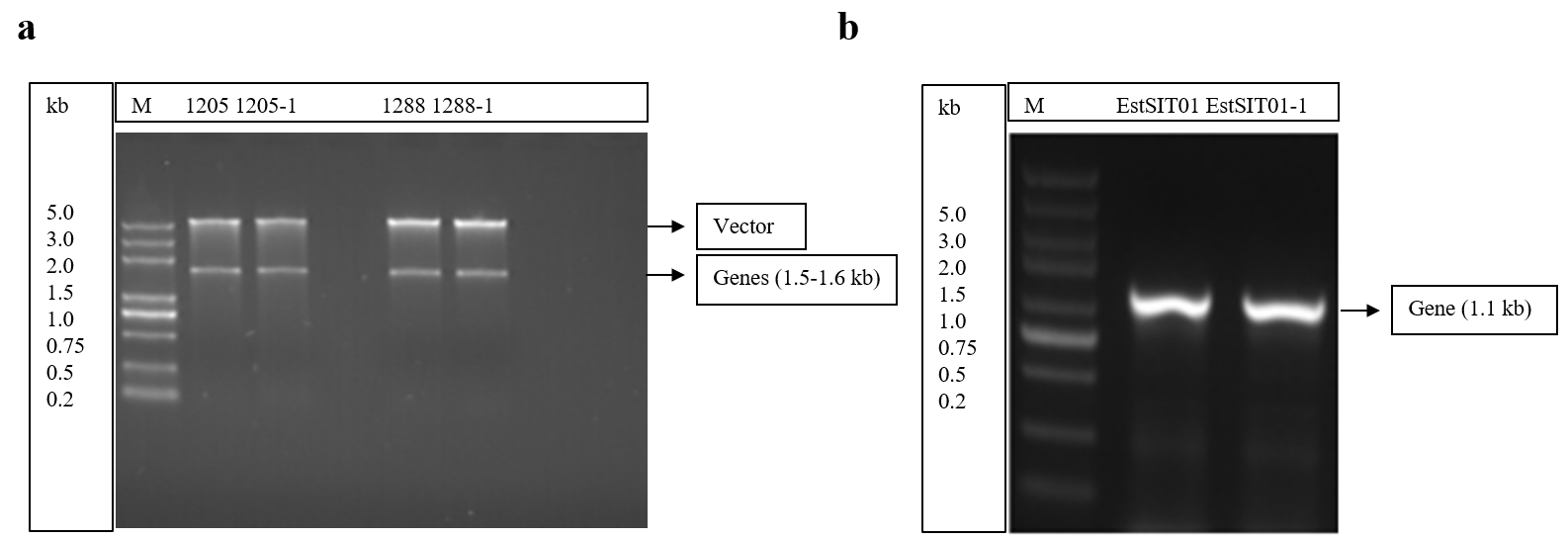
**

**Fig. S1** Plasmids of recombinant *E. coli* of No. 1205 (1563 bp), No. 1288 (1500 bp) and EstSIT01 (1110 bp) were extracted for verification. (**a)** The digestion production of recombinant plasmid: pET21a-1205 (*Nde* I and *Sal* I), pET21a-1288 (*Nde* I and *Xho* I); (**b)** The PCR product of recombinant plasmid pET21a-EstSIT01. The recombinant *E. coli* of 1205, 1288 and EstSIT01 were successfully constructed.


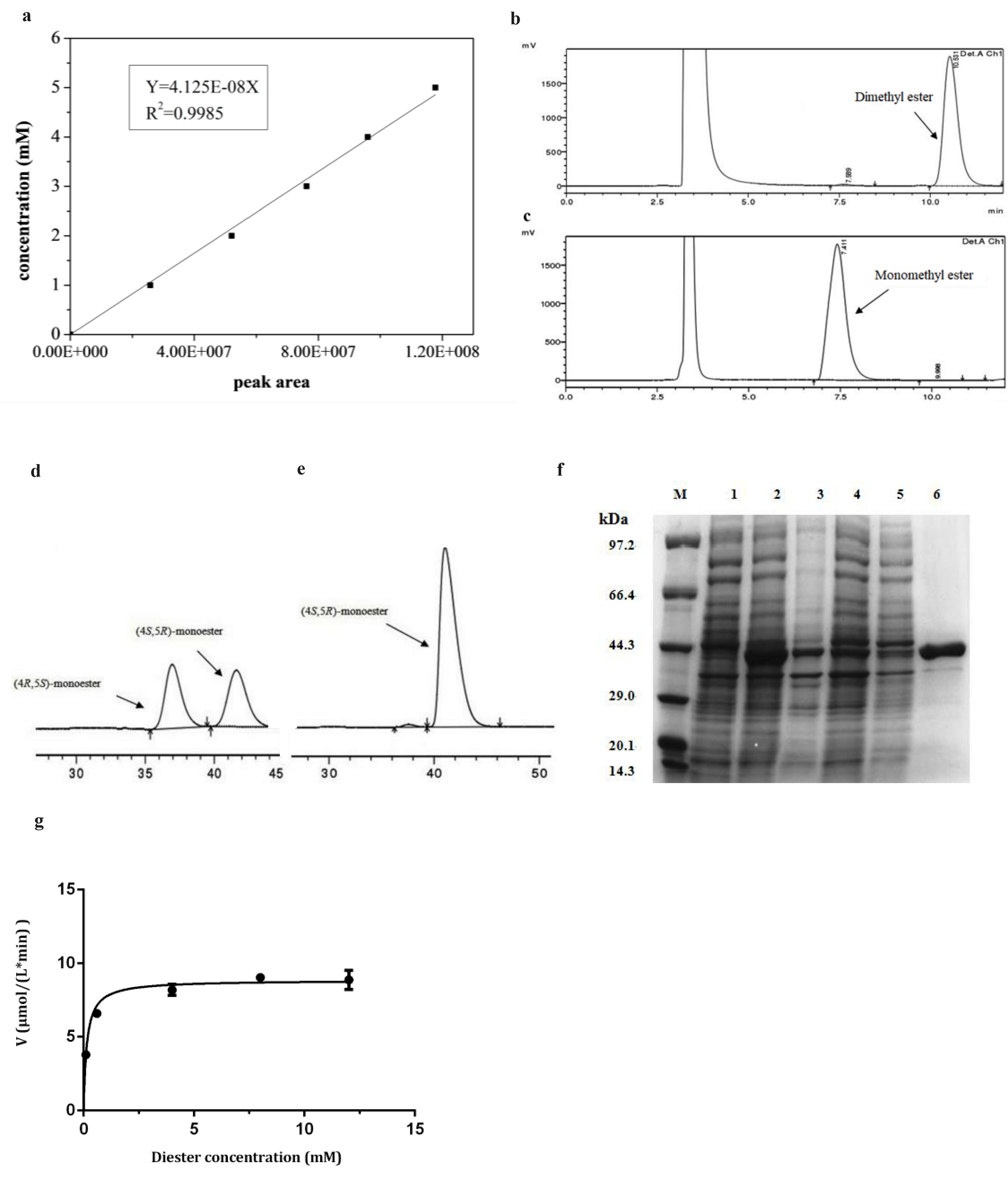


**Fig. S2** Enzymatic synthesis and analysis. **(a)** The calibration curve of (4*S*, 5*R*)-monomethyl ester by HPLC. (**b**) Dimethyl ester before the reaction with C18 column. (**c**) Monomethyl ester after the reaction with C18 column. **(d)** Racemice monomethyl ester prepared by chemical method with Chiralcel OJ-H column. **(e)** (4*S*, 5*R*)-monomethyl ester prepared by enzymatic hydrolysis of dimethyl ester with Chiralcel OJ-H column. **(f)** Purification of EstSIT01. EstSIT01 was expressed in *E. coli* BL21(DE3). The supernatant of the bacterial cell lysate was eluted by a stepwise imidazole gradient. M: Marker proteins, 1: uninduced supernatant, 2: induced supernatant, 3: induced sediment, 4: flow through, 5: 50 mmol/L imidazole, 6: 250 mmol/L imidazole. The weight of EstSIT01 was calculated as 39 kDa. **(g)** Michaelis-Menten plot of EstSIT01.


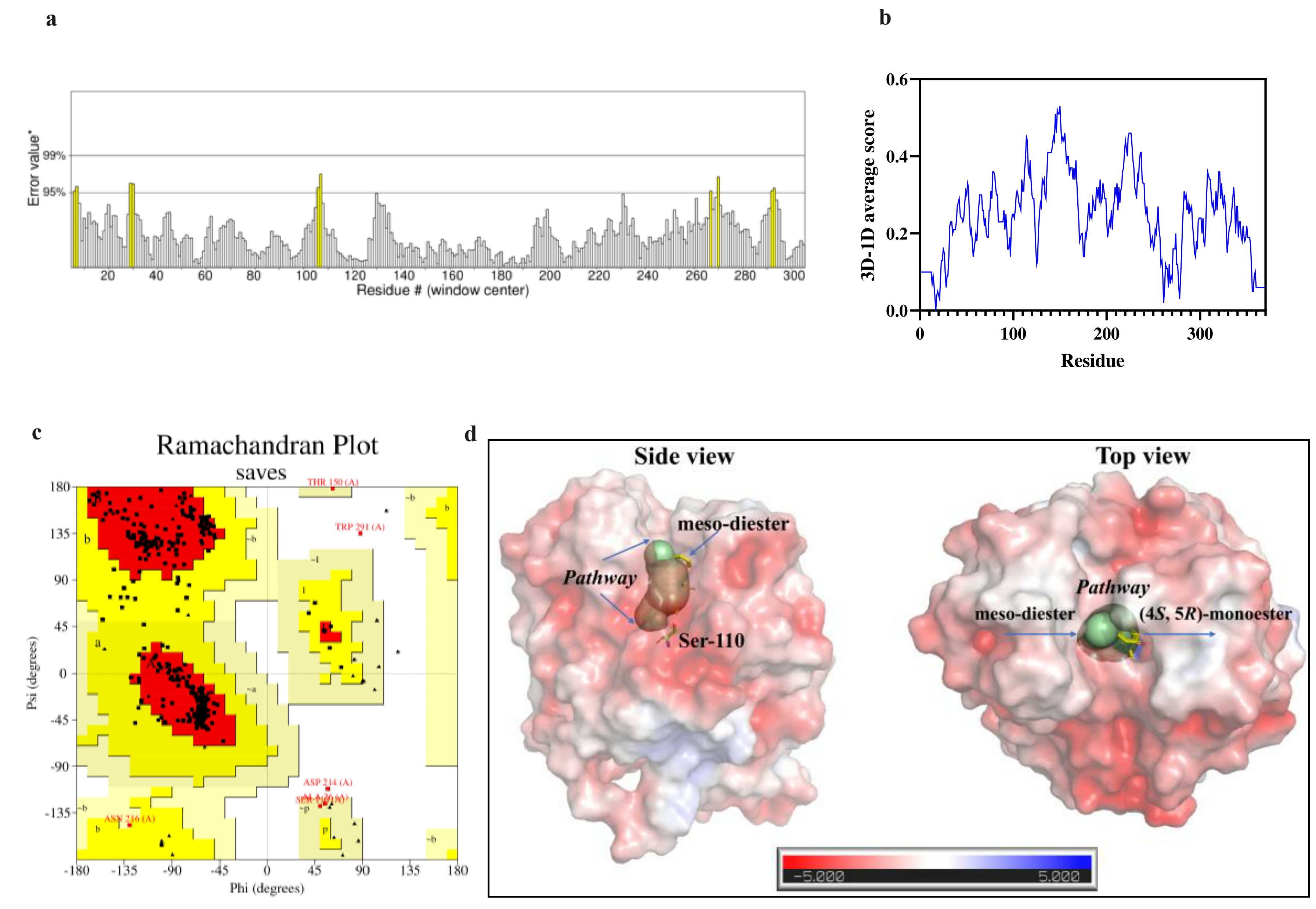


**Fig. S3** Structure prediction and simulation. (**a)** ERRAT: Overall Quality Factor: 95.53. (**b)** VERIFY 3D: 90.27% of the residues have averaged 3D-1D score >= 0.1. **(c)** Ramachandran plot of the modeling result of EstSIT01. **(d)** The pathway from the protein surface to the binding site (Ser110) is identified and contoured with the Caver 3.02 plugin in PyMol.

**Table S1** List of primers

| Enzyme | Forward primer (5’-3’) | Reverse primer (5’-3’) |
| --- | --- | --- |
| ANG84352.1 (1205) | CATATGGCATCCCTCGATGATGGCACCAGCCCCT | GTCGACGAAGGCGAGCGGCGTCTTGCCCCATCCC |
| EstSIT01 | GGAATTCATGACCCTGTTCGACGGCATCACGTCT | CCCAAGCTTGTCGGCGGAGCGGATGATGATCGCCTC |
| ANG84283.1 (No.1288) | CATATGACACCCGTGAACCCTGACCCC | CTCGAGCAGGGCCCGGACCGAGGCGTA |

The digestion sites were underlined, 1205 (*Nde* I and *Sal* I), EstSIT01 (*EcoR* I and *Hind* III), 1288 (*Nde* I and *Xho* I).

**Table S2** Genebank ID of 15 alpha/beta hydrolases and esterases.

| **Alpha/beta hydrolases** | **Esterase** |
| --- | --- |
| ANG84074.1, ANG84216.1, ANG84305.1, ANG86534.1, ANG85477.1, ANG85655.1, ANG85884.1, ANG85956.1,  ANG84115.1 (EstSIT01), | ANG84230.1, ANG84686.1, ANG84283.1 (No.1288), ANG84352.1 (No.1205), ANG86616.1, ANG84916.1 |
